# Chaos May Prevail Without Filial Piety: A Cross-Cultural Study on Filial Piety, the Dark Triad, and Moral Disengagement

**DOI:** 10.3389/fpsyg.2021.738128

**Published:** 2021-10-22

**Authors:** Xiuqing Qiao, Yiqing Lv, Aamer Aldbyani, Qingke Guo, Tianyi Zhang, Minghang Cai

**Affiliations:** ^1^School of Psychology, Shandong Normal University, Jinan, China; ^2^Department of Educational and Psychological Science, Thamar University, Dhamar, Yemen; ^3^Department of Psychological Counseling, Shandong Xinkang Prison, Jinan, China; ^4^Guangxi University and College Key Laboratory of Cognitive Neuroscience and Applied Psychology, Guangxi Normal University, Guilin, China

**Keywords:** dual filial piety model (DFPM), dark triad (DT), moral disengagement, narcissism, Machiavellianism, psychopathy, culture

## Abstract

In traditional Chinese society, filial piety (FP) served as the philosophical foundation of social governance, without which chaos would prevail. It indicates that the function of FP is not limited to family. FP can predict attitudes and behaviors in other social contexts. This study examined the relationship between FP and moral disengagement, and the mediating roles of the dark triad personality, and cultural differences regarding these mechanisms. An online self-report survey was conducted in two different culture groups- university students from China (*N* = 400, 37% male, M_age_ = 20.41, SD age = 2.52) and Islamic countries who are studying in China (*N* = 378, 59.25% male, M_age_ = 24.29, SD age = 4.77). Correlation analysis showed that authoritarian FP was positively associated with moral disengagement among students from China and Islamic countries, while reciprocal FP only negatively correlated with moral disengagement among Chinese students. Moreover, reciprocal FP directly and negatively affected moral disengagement, and did so indirectly through the mediating role of Machiavellianism. However, authoritarian FP directly and positively influenced moral disengagement, and did so indirectly through the buffering role of narcissism. These two parallel mediating models are not affected by culture. Though FP varies from culture to culture, reciprocal FP and authoritarian FP play critical roles in influencing personality and moral development. Reciprocal FP reduces moral disengagement directly and indirectly by weakening Machiavellianism. The role of authoritarian FP is conflicting. It can strengthen moral disengagement, but may also weaken it by deterring the development of the narcissistic personality. The findings enlighten us to view authoritarian FP dialectically. These two parallel mediating models are not affected by culture, indicating the applicability of DFPM in other societies. Future studies are encouraged to involve participants from more divergent countries and cultural backgrounds.

## Introduction

Morality is the foundation of Confucianism. In traditional China, filial piety (FP) plays a primary role in shaping an individual’s moral development. “One cannot successfully pursue the ethical life outside of fulfilling certain familial and social obligation” (Ivanhoe, 2000, p. 17; Yeh, 2006). However, few empirical studies have so far explored the direct relationship between filial piety and moral development. Previous research considers filial piety as the root of Confucian beliefs. The universality of filial piety’s function in other cultures has seldom been investigated. For example, “pietas” in Latin and “sawab” in Islam both mean filial piety to parents and are regarded as one of the most important virtues. Extant literature on filial piety focused on its influences on care giving behavior and aging policy in Confucian-influenced countries, but neglected its role in influencing individuals’ psycho-social functioning (Chen et al., 2016; Bedford and Yeh, 2020). Recently some researchers called for expanding the scope of filial piety studies, both in the research fields and in applications in other cultural backgrounds (Tan et al., 2019; Zhou et al., 2020; Bedford and Yeh, 2021). Yeh and Bedford (2003) have proposed a revised dual filial piety model (DFPM), shifting the focus of conceptualization of filial piety from cultural norms to the inherent structure of the relationship between parents and children. DFPM focuses on two aspects of parent-child interaction in daily life, authoritarian filial piety (AFP) and reciprocal filial piety (RFP). RFP reflects the egalitarian interaction between parents and children based on mutual love and emotional warmth (Chen and Ho, 2012); AFP reflects the social norms requiring children to satisfy their parental demands and is driven by a need for group identification or social belonging (Yeh et al., 2013; Chen et al., 2016; Yeh and Bedford, 2019). The DFPM stresses that the dual mechanisms underlying parent-child relations are universal and not specific to one cultural context, providing a theoretical foundation for filial piety in non-Chinese societies. The current study was designed to explore the link between filial piety and moral disengagement and the mediating roles of dark triad personality traits. Additionally, this study examined the universality of the function of filial piety by comparing students from China and Islamic countries.

### The Relationship Between Filial Piety and Moral Disengagement and Its Cross-Cultural Universality

People sometimes engage in immoral conduct that violates the ethical principles they stick to. However, some people may be psychologically convinced that ethical standards do not apply to themselves or that their destructive behaviors are morally acceptable in a particular situation. This self-defending or self-serving social cognition process is conceptualized as moral disengagement (MD) by Bandura (Bandura, 1999; Moore, 2015). According to Bandura’s theory of moral agency, moral disengagement is a socialization process embedded within specific cultural contexts (Bandura, 2016, p7). This suggests that filial piety belief, as a variable integrating the roles of individuals, society, and cultural norms, can greatly influence moral cognition and moral decision-making (Bedford and Yeh, 2019). As the primary moral agency, parent-children interaction can significantly influence the development of moral disengagement (Campaert et al., 2018). Correspondingly, previous literature shows that inductive discipline and adequate monitoring can reduce children’s moral disengagement, while harsh parenting may lead to greater moral disengagement (Ball et al., 2017; Campaert et al., 2018). Surprisingly, few empirical studies have so far directly investigated the relationship between filial piety and moral disengagement.

In traditional China, disrespect to parents is labeled as immoral because being filial is compulsory. Filial piety emphasizes children’s responsibility to parents (Bedford and Yeh, 2019; Wei and Liu, 2020), which can be manifested in children’s moral cognition (showing deference and obedience to parents and give priority to interests of the family over that of one’s own), moral emotion (showing love, gratitude, respect to parents), and moral conduct (caring for parents and family elders, providing material support). Filial beliefs can influence children’s interpersonal relationships and social roles even after entering adulthood and beyond (Yeh and Bedford, 2019). Empirical studies indicated that the two types of filial piety beliefs have different effects on young adults’ psychological and social adaptation (Chen, 2015). RFP showed a positive effect in enhancing interpersonal relationships, decreasing parent-child conflicts, and increasing academic achievement (Chen and Ho, 2012; Zhou et al., 2020); AFP is significantly associated with maladaptive emotions and behaviors (Yeh, 2006). A recent study found that the parenting style featured by higher levels of rejection, over-protection, and lower level of emotional warmth (all of these overlaps with AFP essentially) has a role in promoting cyber-aggression of postgraduate students in universities through the mediating of moral disengagement (Zhang et al., 2021).

On the contrary, secure parental attachment and high-quality family function, closely associated with RFP, can inhibit moral disengagement (Bao et al., 2015; Mazzone and Camodeca, 2019). Based on the above theorizing, we proposed that AFP and RFP have opposite effects on individuals’ moral disengagement. It should be noted that Chinese filial piety overlaps with family ethics or values in other cultures and filial piety can be regarded as a universal dual mechanism of parent-child interaction beyond ethical norms root in Confucian culture (Bedford and Yeh, 2019, 2021). This laid the foundation for cross-cultural studies of the functions of FP. Consistent with this proposition, the relationships between filial piety and psychological outcomes found in non-Confucian-influenced societies (Bergelson et al., 2015; Toro et al., 2019) replicate those revealed by studies conducted in Chinese societies (2016; Chen, 2015; Zhou et al., 2020). These findings suggest that filial piety can be seen as a universal construct, even if its effects on psychological outcomes differ across cultures (Bedford and Yeh, 2019). Therefore, we supposed that the FP-MD association has cross-cultural stability. Accordingly, this study proposes the following hypotheses:

H1a: RFP is negatively correlated with moral disengagement in both Chinese and Islamic cultures.
H1b: AFP is positively correlated with moral disengagement in both Chinese and Islamic cultures.

### The Dark Triad as Mediators

The term dark triad refers to the constellation of three traits: Machiavellianism, Narcissism, and Psychopathy (Paulhus and Williams, 2002). Machiavellianism can be characterized by a cynical disregard for morality, lack of empathy, and focusing on personal interest and ambition. To maximize personal gain, Machiavellians often manipulate and exploit others (Muris et al., 2017). Narcissism is characterized by a grandiose sense of self-value and a strong need for appreciation and admiration. Narcissists are self-centered and often consider themselves deserving of special treatment (Muris et al., 2017). Psychopathy can be characterized by aloofness, an absence of empathy, remorse or guilt, poor behavioral control, and irresponsibility (Muris et al., 2017). Finally, the dark triad reflects a non-communal, manipulative, exploitative, and self-focused approach to interpersonal relations (Furnham et al., 2013; Miller et al., 2019). The above features suggest that people high in Machiavellianism and psychopathy are more likely to cross moral boundaries to engage in unethical behavior than those scoring low in Machiavellianism and psychopathy (Sijtsema et al., 2019). Individuals high in narcissism may be prone to commit moral transgression because their own interests have been given priority over the interests of others (Egan et al., 2015). Therefore, the dark triad can be considered as an antecedent of moral disengagement.

In the DFPM, RFP, and AFP co-exist within an individual, influencing the development of personality (Bedford and Yeh, 2019). RFP, originated from secure attachment style in parent-child bonds, adequate parental care, and appropriate discipline, can prevent children from manipulating others (Machiavellianism), being self-centered (Narcissism), and acting in the absence of guilt (Psychopathy, see Jonason et al., 2014; Liu et al., 2019). According to life history theory, negative early life experiences, such as harsh parenting and unpredictable parental behavior, may result in pathological personality development (e.g., exploitation, manipulation; Csathó and Birkás, 2018). AFP, which emphasizes obedience and obligations, is more likely to originate from families featured by harsh parenting (Bedford and Yeh, 2019). This suggests that high AFP individuals tend to adapt a fast life history strategy conducive to dark triad personality, while high RFP individuals tend to do the opposite (Jonason et al., 2017; Csathó and Birkás, 2018). Therefore, we propose that filial piety beliefs play essential roles in the development of dark triad personality that is closely associated with unethical decision making (Jonason et al., 2014; Frankenhuis et al., 2016; Csathó and Birkás, 2018; Mazzone and Camodeca, 2019; Abdollahi et al., 2020; Li et al., 2020). Specifically, dark triad traits play mediating roles in the relationship between filial piety and moral disengagement.

As previous literature has suggested, three components of the dark triad may influence moral development in different ways (Egan et al., 2015; Sijtsema et al., 2019; Abdollahi et al., 2020; Kay and Saucier, 2020). We propose that the three dark triad traits serve as multiple parallel mediators in the filial piety-moral disengagement relation. Meanwhile, considering that RFP and AFP are related but distinct constructs, we proposed the following hypotheses:

H2a. The three dark triad traits serve as parallel mediators in the associations between RFP and moral disengagement.
H2b. The three dark triad traits serve as parallel mediators in the associations between AFP and moral disengagement.

### The Current Study

**FIGURE 1:**
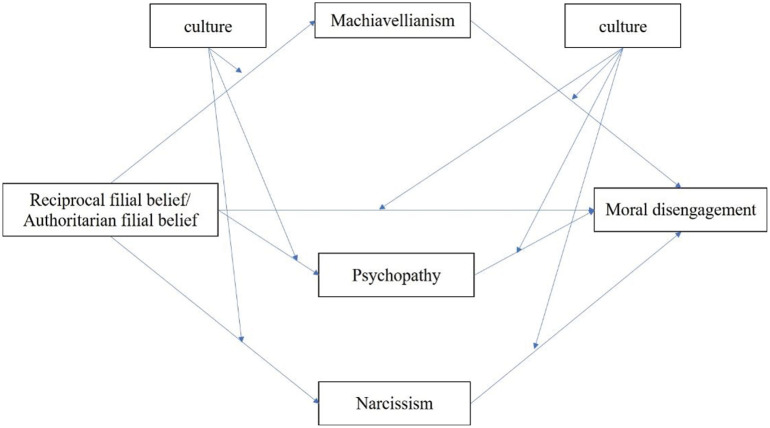
The proposed moderation effects of culture in the mediation models.

Previous studies have mainly focused on parenting styles and parent-child interactions on children’s moral disengagement. There is a literature gap in how filial piety influences moral disengagement. The current study explored whether the dark triad personality mediated the relations between filial piety beliefs and moral disengagement. In addition, we planned to explore whether the above mediation models show cross-cultural stability. There are many similarities and dissimilarities between Chinese culture and Islamic culture (Khalaila, 2010). To our knowledge, no previous studies have compared the mechanism of moral disengagement between the two cultural groups. Therefore, we try to use culture background as a moderating variable to explore whether the above mediation models are different in these two cultural groups. Figure 1 illustrates the conceptual model.

## Materials and Methods

### Participants and Procedures

A total of 807 university students from three countries (China, Indonesia, and Yemen) were recruited. They were required to complete the measures of the dark triad personality, filial piety beliefs, and moral disengagement online. To Indonesia and Yemen students, the English versions of these measures were administrated. To Chinese participants, the Chinese versions were administrated. Participants had informed of the voluntary nature of the investigation and were encouraged to complete all items honestly. Participants were also told that their scores would be kept anonymously and confidentially. Participants were also asked to report their demographic characteristics (e.g., gender, age, family economic status). Participants who omitted at least one questionnaire item, who were Indonesians or Yemenis but reported non-Muslim faith, and who were Chinese but reported religious faith were excluded (16 Chinese participants were excluded on account of their religious beliefs). Finally, we had 778 valid cases (96.41% were valid in total). Of the total number of participants, 400 were students from China (N_female_ = 253, M_age_ = 20.41, SD _*age*_ = 2.52), 378 were international students in China from Islamic countries, Indonesia, and Yemen (Indonesia, *N* = 250, N_female_ = 125, M_age_ = 22.13, SD _*age*_ = 2.61 and Yemen, *N* = 128, N_female_ = 29, M_age_ = 28.51, SD _*age*_ = 5.20). After the administration, participants were thanked and paid 20 yuan for compensation.

### Measures

#### Dark Triad

The 12-item Dirty Dozen scale (DD; Jonason and Webster, 2010) measured participants’ dark triad traits. Each item was rated on a 5-point Likert-type scale ranging from 1 (strongly disagree) to 5 (strongly agree). Example items of three subscales are “I tend to manipulate others to get my way” (Machiavellianism), “I tend to lack remorse” (Psychopathy), and “I tend to want others to admire me” (Narcissism). In the Chinese population, DD has showed good reliability and validity (Geng et al., 2015). In this study, Cronbach’s alpha of the three subscales were 0.72 (Machiavellianism), 0.74 (Psychopathy), and 0.75 (Narcissism) among Chinese participants, and 0.89 (Machiavellianism), 0.88 (Psychopathy), and 0.87 (Narcissism) among participants from Islamic countries.

#### Filial Beliefs

Filial beliefs, including reciprocal filial and authoritarian filial beliefs, were measured by the Filial Piety Scale (FPS; Yeh and Bedford, 2003; Chen, 2014). FPS consists of 16 items, each uses a 6-point scale ranging from 1 (extremely unimportant) to 6 (extremely important). The reciprocal filial belief dimension (e.g., be frequently concerned about my parents’ general well-being) and the authoritarian filial belief dimension (e.g., taken my parents’ suggestions even when I do not agree with them) each includes eight items. The total scores of all items of each dimension were taken to represent the levels of filial beliefs. FPS has shown acceptable reliability and validity in previous research among Chinese samples (Yeh et al., 2013). In this study, Cronbach’s alpha for reciprocal and authoritarian filial beliefs were 0.88 and 0.61 among Chinese participants, and 0.89 and 0.80 among Islamic participants.

#### Moral Disengagement

The 32-item Moral Disengagement Scale (MDS; Bandura et al., 1996) was used to assess eight moral disengagement tactics (e.g., telling small lies is permitted because no one is hurt). The Chinese version of MDS shows good reliability and validity in young adults (Wang et al., 2017). For brevity, only the total score of MDS was used in this study. Participants are instructed to rate each item using a 5-point scale (1 = strongly disagree; 5 = strongly agree). In this study, α coefficients were 0.95 (Chinese participants) and 0.93 (Islamic participants).

### Control Variables

Considering the correlation between gender, age, socioeconomic status, and moral disengagement in previous studies (Bandura, 2016; Alexandra et al., 2021; p. 290; Charalampous et al., 2021), gender, age, and monthly family income were used as control variables to enhance the validity of research findings. The participants were asked to report their monthly family income using a scale with 6 response options, namely, 1000–1999¥, 2000–2999¥, 3000–3999¥, 4000–4999¥, 5000–5999¥, and ≥ 6000¥.

## Results

Harman’s single factor test was conducted to identify common method bias (Podsakoff et al., 2003). All three scales were subjected to exploratory factor analysis, yielding 10 factors with eigenvalue over one, with the first unrotated factor accounting for 25.00% of the total variance, suggesting that the relations among research variables may not be contaminated by common method bias.

### Correlations Among Research Variables

Pearson correlation analysis was conducted to estimate the relationships among research variables. The results (Table 1) showed that AFP was positively correlated with MD among participants from both China (*r* = 0.34, *p* < 0.00) and Islamic countries (*r* = 0.25, *p* < 0.001), while RFP only negatively correlated with MD among Chinese participants (*r* = −0.52, *p* < 0.001). So far, H1a is partially supported and H1b is completely supported. Besides, three dark triad traits, Machiavellianism, Psychopathy, and Narcissism, were all positively associated with MD, regardless the participants were Chinese (*r* = 0.45, 0.24, 0.41, *p*s < 0.001) or Muslims (*r* = 0.63, 0.24, and 0.52, *p*s < 0.001).

**TABLE 1:** Descriptive statistics and Pearson correlations among research variables.

	**1**	**2**	**3**	**4**	**5**	**6**
1 R.F.P	−	−0.14***	−0.36***	−0.02	−0.26***	−0.52***
2 A.F.P	0.63***	−	0.17**	−0.10*	−0.00	0.34***
3 Machiavellianism	−0.15**	0.10	−	0.44***	0.64***	0.45***
4 Psychopathy	0.08	0.16**	0.19***	−	0.58***	0.24***
5 Narcissism	−0.17**	0.09	0.75***	0.23***	−	0.41***
6 M.D.	−0.07	0.25***	0.63***	0.24***	0.52***	−
*Mean (SD)_*Islamic*_*	39.00 (7.03)	33.29 (7.01)	8.79 (4.10)	12.47 (3.90)	8.85 (3.96)	88.92 (19.98)
*Mean (SD)_*China*_*	39.00 (6.57)	25.20 (5.07)	13.06 (4.49)	18.20 (4.71)	14.03 (5.16)	70.27 (22.05)
*F*(1, 776)	0.01	342.29***	190.27***	340.60***	245.27***	152.40***

R.F.P., Reciprocal filial piety, A.F.P., Authoritarian filial piety, M.D., Moral Disengagement. The upper right part of the diagonal of the table is the results of the Chinese sample, and the lower left part is the results of the Islamic sample. N_*Chinese*_ = 400, N_*Islamic*_ = 378. ***p < 0.001, **p < 0.01, *p < 0.05.

### The Relationship Between Filial Piety Belief and Moral Disengagement: Across-Cultural Comparison

#### The Mediation Effects of the Dark Triad

**FIGURE 2:**
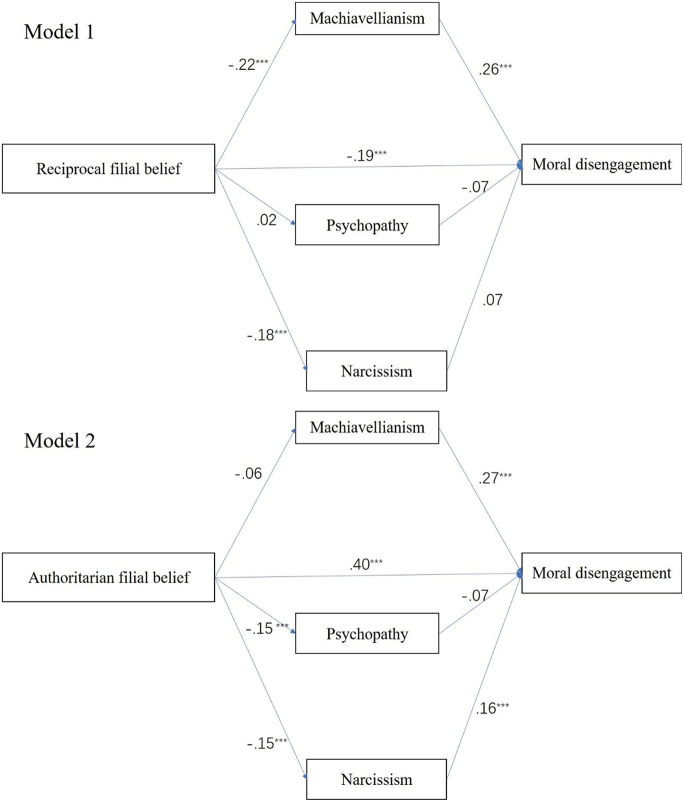
The parallel mediation models of dark triad traits. *N* = 778, ***p < 0.001; Bootstrap = 5,000.

Model 4 (a template for mediation analysis) of PROCESS macro for SPSS (Hayes, 2012) was used to examine the indirect effect of filial piety on moral disengage-ment through three dark triad traits (H2a and H2b). Gender, age, and monthly family income were entered as control variables. As shown in Table 2 and Figure 2 (Model 1), RFP had a significant negative direct effect on moral disengagement (*b* = −0.19, SE = 0.11, *p* < 0.01, 95%CI = [−0.87, −0.44]). And the total mediation effect of the dark triad between RFP and moral disengagement was significant (*b* = −0.07, SE = 0.01, 95%CI = [−0.10, −0.05]). More specifically, the mediation effect via Machiavellianism was significant (*b* = −0.06, SE = 0.01, 95%CI = [−0.09 −0.03]), the mediation effects via psychopathy and narcissism were not significant considering that a2 (R.F.P.→Psychopathy), b2 (Psychopathy→M.D.), and b3 (Narcissiam→M.D.) in Table 2 were not significant (*ps* > 0.05). This suggested that RFP had a direct effect and Machiavellianism played a mediating role in the whole model.

**TABLE 2:** Mediation models of reciprocal filial beliefs (*N* = 778).

**Model 1**	***b***	***SE***	***t***	***95% CI***
R.F.P.→Machiavellianism(a1)	–0.22	0.02	−6.80***	[−0.20, −0.11]
R.F.P.→Psychopathy(a2)	0.02	0.03	0.53	[−0.04, 0.06]
R.F.P.→Narcissism(a3)	–0.18	0.03	−5.56***	[−0.19, −0.09]
Machiavellianism→M.D. (b1)	0.26	0.23	5.40***	[0.79, 1.70]
Psychopathy →M.D.(b2)	–0.07	0.18	–1.68	[−0.67, 0.05]
Narcissism→M.D.(b3)	0.07	0.22	1.34	[−0.13, 0.73]
R.F.P.→M, D. (c′)	–0.19	0.11	−5.95***	[−0.87, −0.44]
Age	0.11	0.19	3.41***	[0.26, 0.97]
Gender	–0.18	1.49	−5.67***	[−11.37, −5.22]
Monthly family income	–0.28	0.43	−8.50***	[−4.55, −2.84]
Mediation effects				
Total	–0.07	0.01		[−0.10, −0.05]
R.F.P.→Machiavellianism→M.D.	–0.06	0.01		[−0.09, −0.03]
R.F.P.→Psychopathy→M.D.	–0.01	0.01		[−0.08, 0.01]
R.F.P.→Narcissism→M.D.	–0.001	–0.003		[−0.01, 0.004]

*R.F.P., Reciprocal filial piety, M.D., Moral Disengagement.*

***p < 0.001; Bootstrap = 5,000.

Model 2 estimated the parallel mediation of dark triad between AFP and moral disengagement (Table 3 and Figure 2). Results showed that AFP had positive direct effect on moral disengagement (*b* = 0.40, SE = 0.10, 95%CI = [1.06, 1.46]) and the total mediation effect was significant (*b* = −0.03, SE = 0.01, 95%CI = −0.06, −0.01]), among them, only the mediation effect of narcissism was significant (*b* = −0.02, SE = 0.01, 95%CI = −0.04, −0.01]).

**TABLE 3:** Mediation models of authoritarian filial beliefs (*N* = 778).

**Model 2**	***b***	***SE***	***t***	***95% CI***
A.F.P.→Machiavellianism (a1)	–0.06	0.02	–1.74	[17.45, 22.89]
A.F.P.→Psychopathy (a2)	–0.15	0.03	−4.16***	[−0.16, −0.06]
A.F.P.→Narcissism(a3)	–0.15	0.03	−4.16***	[−0.16, −0.06]
Machiavellianism→M.D. (b1)	0.27	0.21	6.05**	[0.87, 1.71]
Psychopathy →M.D. (b2)	–0.07	0.17	–1.80	[−0.63, 0.03]
Narcissism→M.D. (b3)	0.16	0.21	3.31***	[0.28, 1.10]
A.F.P.→M.D. (c’)	0.40	0.10	12.41***	[1.06, 1.46]
Age	0.08	0.17	2.59**	[0.11, 0.77]
Gender	–0.12	1.41	−4.18***	[−8.63, −3.11]
Monthly family income	–0.17	0.42	−5.29***	[−3.07, −1.41]
Mediation effects				
Total	–0.03	0.01		[−0.06, −0.01]
A.F.P.→Machiavellianism→M.D.	–0.02	0.02		[−0.04, 0.004]
A.F.P.→Psychopathy→M.D.	0.01	0.01		[−0.00, 0.03]
A.F.P.→Narcissism→M.D.	–0.02	0.01		[−0.04, −0.01]

*A.F.P., Authoritarian filial piety, M.D., Moral Disengagement.*

***p < 0.001, **p < 0.01; Bootstrap = 5,000.

#### Testing for the Moderated Mediation Models: Cross-Cultural Comparison

So far, this study has tested the mediating role of the dark triad. To further explore the possible differences in the mediating models, Model 59 of PROCESS macro for SPSS was adopted, using culture as a common moderator (Hayes, 2015). Gender, age, and monthly family income were entered as control variables. The index of moderated mediation was presented in Table 4. Results showed that all 95% bootstrap confidence intervals (the index of moderation mediation) contained zero, suggesting that the mediation path of filial piety- dark triad traits-moral disengagement did not differ across the two samples.

**TABLE 4:** Culture differences in the mediation effect.

			**Effect**	**SE**	**95%CI**	**INDEX**	**SE**	**95%CI**
Model 3	R.F.P.→Machiavellianism→M.D.	China	–0.17	0.08	[−0.34, −0.03]	–0.03	0.11	[−0.24, 0.19]
		Islamic	–0.20	0.07	[−0.35, −0.07]			
	R.F.P.→Psychopathy→M.D.	China	–0.01	0.01	[−0.04, 0.03]	0.04	0.02	[−0.01, 0.09]
		Islamic	0.03	0.02	[−0.00, 0.07]			
	R.F.P.→Narcissism→M.D.	China	–0.13	0.05	[−0.24, −0.04]	0.09	0.06	[−0.02, 0.21]
		Islamic	–0.04	0.03	[−0.11, 0.01]			
Model 4	A.F.P.→Machiavellianism→M.D.	China	0.12	0.07	[0.01, 0.29]	–0.01	0.10	[−0.20, 0.18]
		Islamic	0.12	0.06	[−0.005, 0.26]			
	A.F.P.→Psychopathy→M.D.	China	–0.01	0.03	[−0.07, 0.04]	0.06	0.04	[−0.01, 0.14]
		Islamic	0.046	0.02	[0.006, 0.103]			
	A.F.P.→Narcissism→M.D.	China	–0.03	0.06	[−0.16, 0.10]	0.05	0.07	[−0.08, 0.19]
		Islamic	0.054	0.02	[0.009, 0.099]			

*R.F.P., Reciprocal filial piety, A.F.P., Authoritarian filial piety, M.D., Moral Disengagement; N_*Chinese*_ = 400, N_*Islamic*_ = 378.*

*Both models were controlled for age, gender, and monthly family income.*

## Discussion

For thousands of years, filial piety has been regarded as the moral foundation for interpersonal relationships in China and other Confucianism-influenced East Asian countries (Bedford and Yeh, 2019). Little empirical research has investigated whether and how filial piety influences individuals’ moral disengagement. More specifically, questions about the mediating mechanisms (i.e., how AFP or RFP predicts moral disengagement) and the universality of this relationship in other cultural contexts remain largely unanswered. Therefore, in this study, we constructed two moderated mediation models to address these questions. The results showed that RFP reduced moral disengagement directly and did so indirectly by weakening Machiavellianism. The role of AFP is complicated, which has a direct strengthening effect on moral disengagement but can weaken it by suppressing the narcissistic personality. Furthermore, these two mediation models were not significantly different across different cultural groups. Besides, there were many interesting findings regarding the function of filial piety in different cultural groups in this study.

We first examined the relationship between AFP/RFP and moral disengagement in China and Islamic countries separately. As expected, AFP is significantly and positively associated with moral disengagement in both two cultural groups. This confirms that AFP can generally be seen as an unfavorable family obligation belief regarding individual moral socialization (Yeh, 2003). But the negative association of RFP and moral disengagement is only significant in the Chinese sample. One of the possible explanations is that, in Confucian societies, parent-child interactions are more important in children’s moral development; while in Islam, moral norms are laid down by Allah Almighty but not human beings (Naeem and Shah, 2013). This can partly explain why the positive effects of RFP on moral responsibility are absent among Muslim participants.

Based on previous findings that the dark triad traits are predictors of moral disengagement (Sijtsema et al., 2019), and poor parent-child relationships contribute to the development of the dark triad (Jonason et al., 2014), this study introduced the dark triad traits as the mediating mechanisms in the link of filial piety and moral disengagement. The two parallel mediation models indicated that individuals with higher levels of RFP, who emphasized personal choices and authentic love to parents (Yeh and Bedford, 2004), are less likely to manipulate others or disregard personal moral standards. AFP, which advocates personal sacrifice and suppression of individual needs to fulfill obligations to parents (Yeh and Bedford, 2004), may be responsible for a higher level of moral disengagement. High-level AFP often represents strict parental demands and a disadvantaged position of children in the family hierarchy (Bedford and Yeh, 2019). In such a raising environment, children are more inclined to shirk responsibility to avoid punishment from parents. When external forces mainly control one’s behaviors, it is impossible to require an individual to take responsibility for his/her behaviors (Bandura, 2016). Surprisingly, we found that AFP could reduce moral disengagement by inhibiting the development of narcissism, suggesting that AFP is not entirely negative, as past findings have suggested (Yeh and Bedford, 2004). AFP means that duties to families come before the interests of an individual. One must suppress personal needs and make sacrifices to enhance the well-being of the whole family. Therefore, a filial individual is less likely to show characteristics of high narcissism, such as “I tend to expect special favors from others.”

The insignificant moderation effect of culture indicates that the association of filial piety and moral disengagement via the dark triad is justifiable among Chinese and Muslim participants. First, as a contextualized personality, filial piety can profoundly affect personality development, which in turn influences moral cognition and behavior even in adulthood. Moreover, this provides empirical support for DFPM, which posits that filial piety should be conceptualized mainly in terms of parent-child relationships but not only culture norms (Bedford and Yeh, 2019). Therefore, the function of filial piety is comparable in China and non-Confucian-influenced countries. As Bedford and Yeh (2021) demonstrated, DFPM can be used as the framework to understand inter-generational and inter-personal relationships in different cultures.

Some interesting findings in this study are noteworthy. More specifically, the endorsement of AFP in the Chinese sample is significantly lower than that in the Islamic sample. There are three reasons to explain this difference. Firstly, previous literature has observed a decrease of AFP in modern Chinese societies with industrialization and urbanization (Yeh and Bedford, 2003). Second, past cross-culture studies supported a stronger endorsement of filial duty among Muslims. For example, Duguet et al. (2016) found that the Muslims show a stronger willingness to host elderly parents than Europeans. Third, previous research has found that filial piety beliefs in Arabic cultures involve seven components (i.e., sacrifice, obligation, respect, face-saving, repay, intergenerational exchange, family unity), most of which fit the definition of AFP (Khalaila, 2010). Therefore, it is plausible that the international students from Islamic countries scored higher in AFP than Chinese college students. In addition, we found opposite results regarding the correlations between AFP and RFP in two culture groups. Among Chinese participants AFP and RFP were negatively correlated. This can be interpreted as the effect of modernization and industrialization, which considerably changed Chinese people’s values and beliefs. More and more Chinese people endorse RFP but refuse AFP (Yeh et al., 2013). That is, they yearn for mutual affection and intimacy (RFP) in parent-child relationships and oppose traditional teachings requiring individuals to suppress their needs for the sake of collective interests. Among Muslim participants, however, AFP and RFP were positively correlated. This may reflect the fact that discipline and psychosocial well-being of the children are both highly valued in Islamic cultures. Children are strictly disciplined (AFP) to cultivate obedience to parents and God; meanwhile, the Qur’an, the holy book of Muslims, also requires parents to ensure the development of good psychosocial functioning of their children, which needs a positive parent-child interaction (RFP). This suggests that AFP and RFP are closely connected in Islamic countries (see Oweis et al., 2012; Riany et al., 2017).

It is noteworthy that all the dark triad traits were significantly higher, but moral disengagement was significantly lower in Chinese relative to Muslim participants. This is consistent with Jonason et al. (2020) who found that narcissism, Machiavellianism, and psychopathy of China (4.41, 2.83, and 2.55) were generally higher than Egypt (4.14, 2.13, and 2.43), and Indonesia (3.72, 2.66, and 2.80), two Muslim countries. We have no evidence regarding the difference in moral disengagement scores between Chinese and Muslim students and its cultural implications. We hope future studies can address this issue. Another unexpected finding inconsistent with previous studies (e.g., Egan et al., 2015; Sijtsema et al., 2019) is that narcissism significantly affected moral disengagement. Specifically, in this study, narcissism is not only positively related to moral disengagement but also played a mediating role between AFP and moral disengagement. This can be accounted for by cultural differences between the Eastern and Western societies. Most of the previous studies are conducted in Western individualistic societies, where there is a popularity of cultural products eliciting narcissism, such as song lyrics, reality television and advertisements (Twenge and Campbell, 2009), narcissism is accepted and even encouraged in such situations. Therefore, the different findings on the relation between narcissism and moral disengagement can be accounted for by how strongly the individualistic value is endorsed across cultures. High individualistic individuals tended to make internal attributions when they were asked to explain social events (Oyserman et al., 2002). They tend to assume social responsibilities rather than shirk them. However, in societies that attach great importance to collective goals, such as Islamic countries and China, narcissism is more strongly indicative of selfishness and irresponsibility thus has a stronger relationship with moral disengagement. Nevertheless, these comparable findings in Chinese and Muslim participants illustrate that filial piety is rooted in parent-child interactions rather than behavioral norms shaped by culture (Bedford and Yeh, 2019). Moreover, these findings suggest that family obligation beliefs continued to affect on moral cognition, emotions, and behaviors even after the children enter adulthood and beyond (Wei and Liu, 2020).

## Limitations and Future Directions

Several limitations should be addressed. Filial piety originates from parent-child interaction and can be indicated by children’s perception of family obligation and duty to host the elderly parents (Schwartz et al., 2010). It can be considered as a family environmental factor that can shape personality development. Previous literature has explored a broad range of psychosocial outcomes of the dark triad personality, but few studies have investigated its antecedent (Furnham et al., 2013). This study found that RFP can prevent the development of Machiavellianism and narcissism regardless of cultural contexts. However, no conclusion can be drawn on which filial piety belief can prevent psychopathy. Future research may consider conducting replication studies or using a longitudinal design to confirm the stability of these relationships. This study revealed culturally universal and specific aspects of filial piety beliefs. Relevant findings should be treated with caution because this study involved only young adults from Chinese and Islamic societies who are well educated. For instance, a study found that the two dimensions of filial piety are significantly related to age among participants ranging from 20 to 69 years old (Yeh et al., 2013). Furthermore, nationally representative samples with more divergent cultural backgrounds are expected to be included in future studies.

In many societies, filial obligations imposed on females are quite different from those on males (Brasher, 2021). This suggests that the effects of filial obligations on psychosocial outcomes may be conditioned by gender (Duguet et al., 2016; Wang et al., 2016; Szabó and Jones, 2019). However, this study has not addressed the moderating role of gender in the relationships among research variables because it is beyond our research scope. Finally, filial piety was operationalized in this study as a contextualized personality trait (namely a continuous variable) but not personality type (namely a categorical variable). According to Yeh and Bedford (2004), there are four filial piety types (high reciprocal-low authoritarian; low reciprocal-high authoritarian; low on both, and high on both). Future studies are encouraged to use a larger sample size to explore whether these four filial piety types are differently associated with personality and moral development. In so doing, the interaction between RFP and AFP may be revealed more clearly.

## Conclusion

Using samples from Chinese and Islamic cultures, we introduced filial piety as an antecedent of the dark triad personality and moral disengagement. Results supported the direct positive effect of RFP on reducing moral disengagement and the mediating effect of Machiavellianism. In contrast, the role of AFP is conflicting. It directly strengthens moral disengagement and concurrently weakens moral disengagement by suppressing Narcissistic personality, enlightening us to view AFP dialectically. In addition, due to that, the effects of filial piety on moral disengagement were considerably consistent across two cultural groups, the applicability of DFPM was empirically supported to a large extent. Future studies are encouraged to involve participants from more divergent countries and cultural backgrounds.

## Data Availability Statement

The raw data supporting the conclusions of this article will be made available by the authors, without undue reservation.

## Ethics Statement

The studies involving human participants were reviewed and approved by the Institutional Review Board (IRB) at Shandong Normal University. Written informed consent for participation was not required for this study in accordance with the national legislation and the institutional requirements.

## Author Contributions

XQ and QG designed the research and wrote the manuscript. YL, TZ, MC, and AA collected and analyzed the data. All authors listed have made a substantial, direct and intellectual contribution to the work, and approved it for publication.

## Conflict of Interest

The authors declare that the research was conducted in the absence of any commercial or financial relationships that could be construed as a potential conflict of interest.

## Publisher’s Note

All claims expressed in this article are solely those of the authors and do not necessarily represent those of their affiliated organizations, or those of the publisher, the editors and the reviewers. Any product that may be evaluated in this article, or claim that may be made by its manufacturer, is not guaranteed or endorsed by the publisher.
